# Case Report: Successful endoscopic treatment of gastric outlet obstruction due to duodenal persimmon phytobezoar

**DOI:** 10.3389/fmed.2026.1732300

**Published:** 2026-02-18

**Authors:** Zong-jing Hu, Yan Wang, Cui-mei Ma, Hui-hui Zhou, De-huai Jing

**Affiliations:** Department of Gastroenterology, Affiliated Hospital of Jining Medical University, Jining, Shandong, China

**Keywords:** duodenal bezoar, elderly patient, endoscopic fragmentation, endoscopic instruments, gastric outlet obstruction

## Abstract

Gastric outlet obstruction (GOO) is a clinical syndrome caused by mechanical impediment to gastric emptying. Bezoars account for < 0.4% of all GOO cases. Of these, duodenal bezoars—particularly those obstructing the narrow duodenal bulb—are exceptionally rare. Duodenal persimmon phytobezoar-induced gastric outlet obstruction (GOO) is particularly distinctive in elderly patients, with only a handful of relevant case reports documented in the literature. We present the case of successful endoscopic fragmentation in an 88-year-old female with GOO caused by a persimmon bezoar located in the duodenal bulb. Abdominal CT and upper endoscopy showed a giant bezoar nearly completely obstructing the duodenal lumen. After 3 days of failed chemical dissolution with sodium bicarbonate solution and Coca-Cola, she underwent two endoscopic fragmentations using simple and readily-available endoscopic instruments, such as rat-tooth forceps, snares, and baskets. More than ten persimmon seeds were extracted during the procedure. Upper endoscopy was repeated and revealed that the bezoar had disappeared, with two necrotic pressure ulcers noted in the duodenal bulb. The patient recovered well and was discharged 4 days following the second endoscopic fragmentation. The patient remained asymptomatic during 3 months of follow-up. Phytobezoars are rarely found in the duodenum, as in the case of this patient. This case highlights the feasibility and efficacy of endoscopic therapy for duodenal bezoar-induced GOO, especially in elderly patients. Furthermore, the use of simple, readily-available endoscopic instruments (rat-tooth forceps, snares, and baskets) makes this approach particularly suitable for use in resource-limited settings.

## 1 Introduction

Gastric outlet obstruction (GOO) is caused by diseases that mechanically impede gastric emptying. Bezoars, concretions of undigested or partially digested material in the gastrointestinal tract, are a rare entity and GOO due to duodenal bezoar is an uncommon presentation (1). The reported incidence is < 0.4% in the general population (2). Though rare, bezoars can cause significant morbidity, including bleeding, obstruction, perforation, or fistulization (3). Phytobezoars, resulting from ingestion of vegetable and plant material of certain types, are indigestible masses trapped in the gastrointestinal tract (4). The most common cause of this type of bezoar is persimmons. Clinically, phytobezoars can be treated by chemical dissolution, endoscopy or conventional surgery. we reported the case of a large duodenal persimmon bezoar in an 88-year-old woman who presented with pyloric obstructive symptoms and was successfully treated with endoscopic fragmentation. In the field of endoscopic therapy, although a variety of advanced instruments are available, not all endoscopy centers are fully equipped with them. This case report demonstrates the successful use of simple and readily available endoscopic devices such as rat-tooth forceps, snares and baskets to relieve GOO caused by persimmon phytobezoar in an elderly patient, offering a feasible treatment option for this population, particularly in low-resource settings.

## 2 Case description

**Figure 1:**
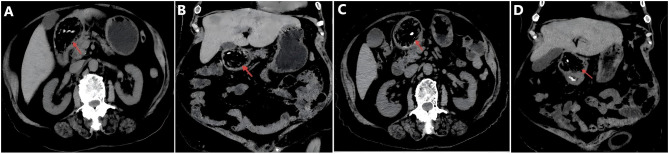
Abdominal computed tomography images. **(A, B)** Axial and coronal CT images showing a large-sized bezoar in the duodenal bulb. **(C, D)** Axial and coronal CT images after 3 days of chemical dissolution treatment.

**Figure 2:**
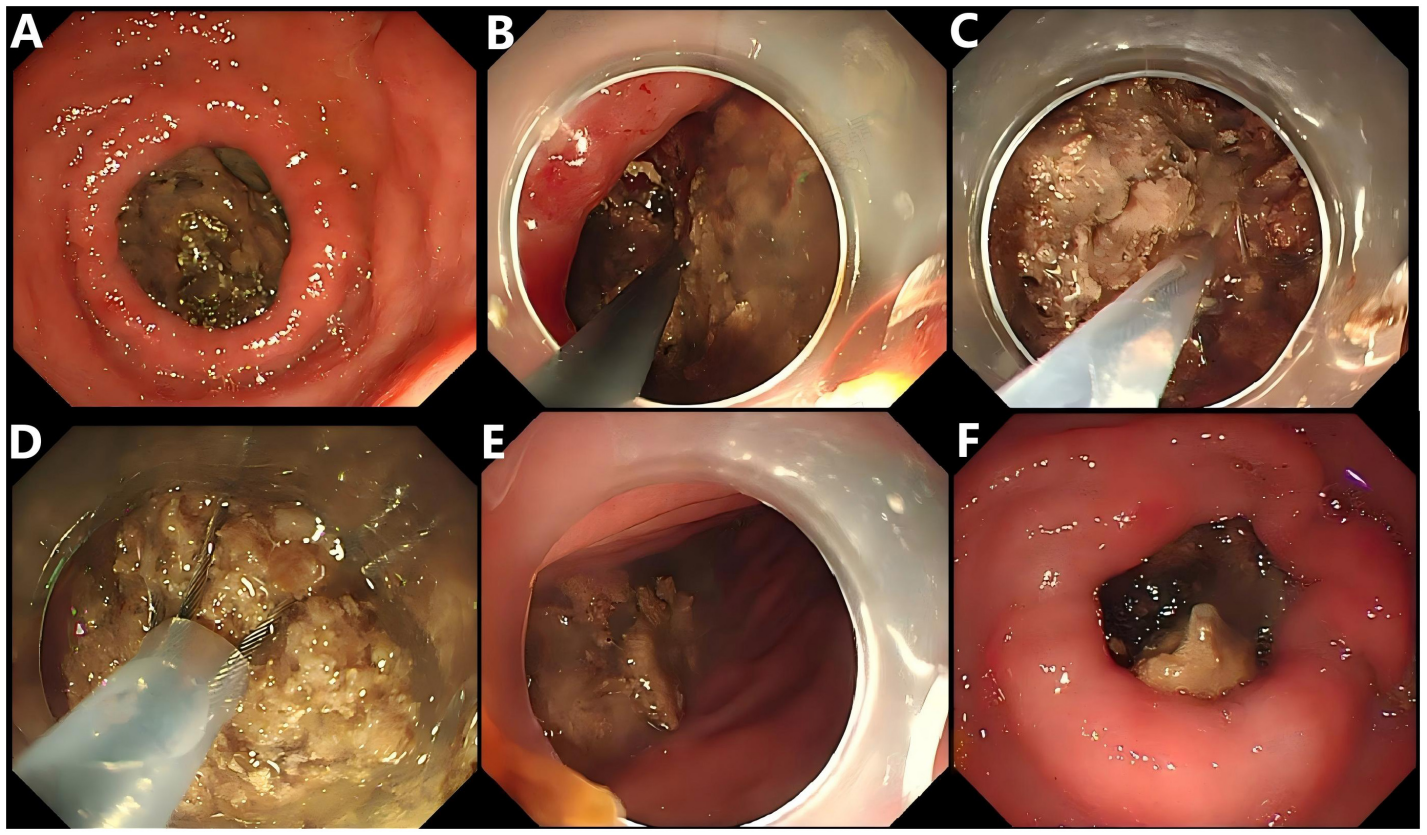
**(A)** Upper endoscopic view of the duodenal bezoar which caused the obstruction of the pyloric canal. **(B, C, D)** Endoscopic images of the fragmentation process of a persimmon phytobezoar using combined application of foreign body forceps, snare, and basket. **(E)** Snare and basket were employed to relocate most of the bezoar into the gastric cavity. **(F)** Upper gastrointestinal endoscopic view taken 3 days after endoscopic fragmentation, showing the bezoar has decreased in size.

**Figure 3:**
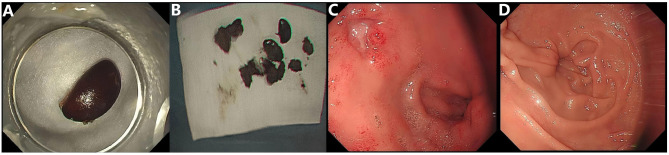
**(A, B)** Extracted persimmon seeds. **(C, D)** Upper gastrointestinal endoscopic view taken 3 days after the second endoscopic fragmentation, showing the bezoar had disappeared and necrotic pressure ulcers were observed in the duodenal bulb.

An 88-year-old woman was admitted to the hospital due to upper abdominal discomfort lasting for half a month, which worsened over the past 3 days and was accompanied by vomiting. The onset of her symptoms was preceded by the consumption of persimmons. She had no significant past medical history, surgical history, or notable family history. On physical examination, she appeared ill and weak. Her abdomen was soft without tenderness. Laboratory tests revealed mild leukocytosis (10.8 × 10^9^/L), hypokalemia (3.2 mmol/L), and hyponatremia (128 mmol/L). Abdominal computed tomography (CT) showed a large-sized (6.0 cm × 4.0 cm) bezoar in the duodenal bulb, which had caused gastric dilatation (Figures 1A, B). Based on the patient's history of persimmon ingestion and abdominal CT findings, a preliminary diagnosis of gastric outlet obstruction caused by duodenal persimmon bezoars was made. She was treated with fasting, fluid and electrolyte replacement, and gastric decompression. In addition, sodium bicarbonate solution and Coca-Cola was infused through the nasogastric tube for stone dissolution, and pantoprazole was administered intravenously. Follow-up abdominal CT after 3 days of chemical dissolution therapy still showed a bezoar in the duodenal bulb (Figures 1C, D). Upper endoscopy showed a giant yellowish-brown bezoar nearly completely obstructing duodenal lumen (Figure 2A). Thus, the diagnosis of duodenal persimmon phytobezoar-induced GOO was confirmed. After obtaining informed consent from the patient, endoscopic fragmentation was performed under general anesthesia with non-endotracheal intubation and monitoring (Figures 2B–D). Due to the bezoar being impacted in the duodenal bulb, the snare could not be deployed properly to ensnare it. Therefore, We utilized rat-tooth forceps to fragment the bezoar piece by piece, gradually exposing the duodenal bulb lumen. Subsequently, the snare was used to further fragment the bezoar. Additionally, the snare and basket were employed to relocate most of the bezoar into the gastric cavity, where they were subsequently fragmented with the snare. Postoperatively, sodium bicarbonate solution was infused into the gastric cavity for bezoar dissolution (Figure 2E). A follow-up upper endoscopy 3 days after the first fragmentation revealed a reduction in the duodenal bezoar size (Figure 2F). A second endoscopic fragmentation was performed using the same technique, during which more than ten persimmon seeds were extracted (Figures 3A, B). Another follow-up upper endoscopy performed 3 days after the second endoscopic fragmentation revealed that the duodenal bezoar had disappeared. A necrotic pressure ulcer was identified on the anterior wall and the lesser curvature of the duodenal bulb, respectively (Figures 3C, D). The patient's diet was advanced, and she was discharged on the fourth day after the second endoscopic fragmentation with full recovery. The patient remained asymptomatic during 3 months of follow-up.

Ethical approval for this case was obtained from the Medical Ethics Committee of the Affiliated Hospital of Jining Medical University. Written informed consent for the publication of anonymized information was obtained from the patient and their authorized representative.

## 3 Discussion

Gastric outlet obstruction (GOO) is an uncommon but potentially serious condition with multiple etiologies, which can be benign or malignant. Prior to the 1980s, peptic ulcer disease was the most common cause of GOO, accounting for up to 90% of cases. However, the advent of Helicobacter pylori eradication regimens and the widespread use of proton pump inhibitors have drastically reduced the incidence of ulcer-related stenosis, leading to malignancy becoming the primary etiology in 50%−80% of current GOO cases (5). Bezoars represent a rare etiology of GOO, arising from the accumulation of foreign material resistant to digestion and gastric emptying (6). This case of an 88-year-old woman with GOO caused by a giant duodenal persimmon phytobezoar enriches the clinical literature on this rare entity. Additionally, it provides critical insights into the diagnosis, treatment, and perioperative management of the condition.

Bezoars are classified according to their composition into phytobezoar, trichobezoars, lactobezoar, mixed medication bezoars and food bolus bezoars (7, 8). The persimmon phytobezoar is a type of phytobezoar caused by ingestion of persimmons (9). Persimmons contain high levels of fiber and shibuol (a tannin), which polymerizes with gastric juice to form diospyrobezoars (9). The research by Hemmasi et al. (3) indicates that the most frequent risk factors of bezoar formation were history of gastric surgery (25.2%), diabetes mellitus (21.3%), hypothyroidism (15.5%), trichophagia (5.8%), and anxiety disorders (2.9%), respectively. In patients without prior gastric surgery or dysmotility, bezoar formation is postulated to develop from changes in dietary habits (10). Elderly individuals are also susceptible to bezoar formation because of their decreased acid, decreased gastrointestinal motility, and a decreased ability to chew (11). Our patient had no prior surgical or psychiatric history. We believed the risk factors include impaired gastric motility and overindulgence of persimmons. Clinical manifestations of persimmon phytobezoar vary from no symptoms to acute abdominal distress including dyspepsia, abdominal pain, and vomiting and nausea. Major complications of bezoars include intestinal obstruction, gastric perforation, gastric ulcer, gastritis, and even gastrointestinal hemorrhage could be seen (12). The diagnosis of duodenal bezoar-induced GOO requires a multimodal approach: clinical suspicion based on symptomology and dietary history, radiological confirmation via abdominal computed tomography (CT), and definitive visualization through upper endoscopy. In our case, abdominal CT initially identified a large hypodense mass in the duodenal bulb with associated gastric dilatation, while upper endoscopy confirmed near-complete obstruction of the duodenal lumen by a giant bezoar. GOO caused by duodenal bezoar is relatively rare, and careful differentiation should be made from other causes of GOO in the elderly population, such as pancreatic cancer, duodenal stenosis, or gallstone ileus.

Bezoar-induced GOO is rarely reported (13, 14), but when it occurs, it mimics pyloric obstruction and causes substantial patient distress. Management is individualized based on the patient's age, comorbidities, bezoar characteristics (size, location, consistency), and local resource availability. Initial supportive measures include fluid and electrolyte replacement (critical in our patient with hypokalemia and hyponatremia), gastric decompression, and proton pump inhibitor administration to reduce gastric acid secretion. Currently treatment options for bezoars include observation, chemical dissolution, and endoscopic fragmentation, surgical treatment via laparotomy or laparoscopy is preferred after a failed conservative procedure (14). In our case, chemical dissolution failed after 3 days. This outcome may be attributed to three key factors: the bezoar's large size, firm consistency, impaction in the narrow duodenal bulb, and the limited penetration of dissolving agents. Given the patient's advanced age and the high morbidity associated with open surgery, endoscopic fragmentation emerged as the optimal minimally invasive treatment strategy. A variety of endoscopic modalities and instruments are used for bezoar fragmentation, including water flushes, directsuction, large polypectomy snare, biopsyforceps, electrosurgical knife, mechanical or extracorporeal lithotripsy (15). Additionally, more specialized techniques such as laser disruption, simple nylon rope lithotripsy have been reported in the literature (16, 17). However, not every endoscopy center is fully equipped with these techniques. Our case highlights the feasibility and efficacy of using simple, readily accessible endoscopic instruments through a stepwise, combined approach. The bezoar was tightly impacted in the narrow duodenal bulb, precluding initial snare deployment. We first used rat-tooth forceps for piecemeal fragmentation to expose the lumen. Snares were then used to reduce fragment size further, and baskets relocated most fragments to the gastric cavity, where the larger operating space allowed more efficient snare fragmentation. Our experience suggests that initial failure of endoscopic management does not warrant immediate abandonment of this approach. Provided the patient remains clinically stable, repeated endoscopic sessions rather than prompt surgical referral can achieve complete bezoar resolution.

This case offers three distinct clinical highlights that address gaps in the existing literature. First, the bezoar's unusual location: most persimmon phytobezoars stay in the stomach; however, this giant bezoar was in the duodenal bulb, an area with a narrow opening and little ability to stretch. This made endoscopic treatment technically challenging, so our successful result is especially notable. Second, the universal accessibility of the endoscopic instruments used in this case equipped in over 95% of secondary hospital endoscopy centers in low-resource regions (18), makes the stepwise fragmentation protocol replicable without relying on specialized devices (e.g., Ho:YAG laser or nylon rope lithotripsy). Third, managing elderly patients with bezoar-induced gastric outlet obstruction (GOO): older adults are more at risk of anesthesia-related problems, surgical site infections, and longer recovery times. Compared with traditional surgery, endoscopic therapy has the advantages of minimal invasiveness, faster recovery, and fewer complications, making it particularly beneficial for elderly patients. Our patient recovered smoothly (discharged 4 days after the second endoscopy) and had no complications during 3 months of follow-up. This supports the use of this approach. While the stepwise fragmentation strategy using conventional endoscopic instruments has demonstrated feasibility and efficacy in this case, it is not without potential risks. The duodenal bulb was the site of bezoar impaction in this case, characterized by luminal narrowing, thin mucosa, and proximity to critical structures such as the pancreatic duct and common bile duct openings. Conventional endoscopic instruments may cause mucosal injury, bleeding, or perforation during lithotripsy or manipulation. Maintaining clear endoscopic visualization throughout the procedure is essential. Adjust the endoscope angle to target the bezoar rather than the mucosa, avoiding blind manipulation. Continue proton pump inhibitor therapy postoperatively to suppress gastric acid secretion and promote mucosal repair. Monitor for signs of severe injury (e.g., hematemesis, melena, worsening abdominal pain). During fragmentation, dislodged bezoar fragments (especially those >1 cm in diameter) may migrate distally, obstructing the jejunum or ileum, particularly in patients with reduced intestinal motility (common in the elderly). Literature reports indicate that the incidence of fragment-induced intestinal obstruction during endoscopic bezoar management ranges from 3% to 5%, with higher rates observed in cases involving large, hard bezoars (15, 18).

Given that our elderly patient was already 88 years old, apart from the anesthetic risks during endoscopic fragmentation, we should also take note of several age-related factors: First and foremost, a comprehensive preoperative assessment is required. Elderly patients are often complicated by underlying comorbidities (e.g., cardiovascular, renal, or pulmonary diseases). Even in the absence of a significant medical history (as in our case), preoperative evaluation is crucial for identifying potential contraindications and optimizing perioperative safety (19). Elderly patients have impaired renal concentrating capacity, which increases their risk of fluid overload or dehydration. Precise fluid replacement is crucial for correcting electrolyte disorders while avoiding the induction of heart failure. Long-term follow-up and patient education are indicated. Persistent gastrointestinal motility impairment increases recurrent bezoar risk in the elderly. Furthermore, follow-up included advising avoidance of high-tannin (persimmons, hawthorns) and high-fiber foods, thorough chewing, and adequate hydration. Patient education plays a vital role in prevention. As highlighted by our patient, rural communities often lack awareness of the link between excessive persimmon consumption and bezoar formation. This case demonstrates that conventional endoscopic tools can be effectively combined to address complex bezoars. Abdominal CT is a cost-effective imaging modality that provides critical information on bezoar size, location, and degree of obstruction, enabling the formulation of a targeted endoscopic strategy. Preoperative gastric decompression and electrolyte correction improve patient tolerance to the procedure. Despite the successful outcome, this case has limitations. As a single-case report, our findings may not be generalizable to all patients with duodenal bezoars. The bezoar's hard consistency and unusual location required two endoscopic sessions, which may not be feasible for patients with poor clinical stability or limited access to healthcare. Additionally, this study did not assess long-term gastrointestinal motility in the elderly patient, which may affect the risk of recurrence. Future studies with larger sample sizes are needed to validate the efficacy of conventional instrument-based endoscopic fragmentation in low-resource settings (18).

In conclusion, this case demonstrates that giant duodenal persimmon bezoars in elderly patients can be safely and effectively managed with endoscopic fragmentation using simple, readily available instruments. The insights gained from this case, particularly those pertaining to resource optimization and the management of elderly patients, offer valuable guidance for clinicians encountering analogous clinical scenarios, especially in resource-limited settings. Endoscopic therapy should be recommended as the first-line intervention for bezoar-induced gastric outlet obstruction (GOO) in elderly patients, serving as a minimally invasive alternative to surgery with favorable clinical outcomes.

### 3.1 Patient perspective

During the 3-month follow-up, the patient expressed great relief that her abdominal discomfort and vomiting had completely resolved, allowing her to resume a normal diet and daily activities. She strongly preferred the minimally invasive endoscopic intervention over open surgery. Additionally, the patient noted that residents in her rural community lack awareness that persimmon consumption may induce gastric bezoars. Thus, she has taken the initiative to disseminate her experience, advocating the following recommendations: avoid ingesting persimmons and other indigestible foods; if such foods are consumed, oral Coca-Cola may be used as an adjunctive measure; and seek immediate medical attention if any gastrointestinal discomfort develops.

## Data Availability

The original contributions presented in the study are included in the article/supplementary material, further inquiries can be directed to the corresponding author.
